# An epidemiological investigation to reconstruct a probable human immunodeficiency virus -1 transmission network: a case report

**DOI:** 10.1186/s13256-015-0717-2

**Published:** 2015-11-03

**Authors:** Sara Serafino, Eleonora Cella, Claudia Montagna, Eugenio Nelson Cavallari, Pietro Vittozzi, Alessandra Lo Presti, Marta Giovanetti, Laura Mazzuti, Ombretta Turriziani, Giancarlo Ceccarelli, Gabriella d’Ettorre, Vincenzo Vullo, Massimo Ciccozzi

**Affiliations:** 1 Department of Public Health and Infectious Diseases, Sapienza University of Rome, Rome, Italy; 2 Department of Infectious Parasitic and Immunomediated Diseases, Reference Centre on Phylogeny, Molecular Epidemiology and Microbial Evolution (FEMEM)/Epidemiology Unit, National Institute of Health, Rome, Italy; 3 Department of Molecular Medicine, Laboratory of Virology, Sapienza University of Rome, Rome, Italy; 4 Department of Biology, University of Rome Tor Vergata, Rome, Italy; 5 University of Biomedical Campus, Rome, Italy; 6 Epidemiology Unit, Department of Infectious, Parasitic and Immune-Mediated Diseases, Istituto Superiore di Sanità- V.le Regina Elena, 299 – 00161 Roma, Italy

**Keywords:** Case report, HIV-1, Phylogeny

## Abstract

**Background:**

Recently published studies have highlighted the importance of phylogenetic and phylodynamic analyses in supporting epidemiological investigations to reconstruct the transmission network of human immunodeficiency virus. Here, we report a case of sexual transmission of human immunodeficiency virus type 1 between a man and a woman that marks once more the importance of a tightened collaboration between phylogeny and epidemiology.

**Case presentation:**

We describe a case of human immunodeficiency virus type 1 subtype B transmission in a stable Caucasian heterosexual couple. The man was 30 years old and the woman was 21 years old at the time of their presentation to the Department of Public Health and Infectious Diseases of the University of Rome “Sapienza”. The couple reported a history of drug abuse.

**Conclusion:**

Phylogenetic analysis is a powerful technique that if properly used can prove valuable in research investigations. In the case presented here, a phylogenetic analysis alongside epidemiological evidence allowed us to determine the most probable source of the human immunodeficiency virus infection. The dated tree allowed us to date the transmission event, the time point, and the direction of transmission based on the phylogeny, which agreed with the presumptive time of infection determined from clinical history-taking.

## Background

The human immunodeficiency virus (HIV) is characterized by great genetic heterogeneity driven by several factors, such as the lack of proofreading ability of the reverse transcriptase [1, 2], the rapid turnover of HIV-1 *in vivo* [3], host selective immune pressures [4], and recombination events during replication [5].

The majority of HIV-1 strains cluster within a large group called M (for Main), which includes nine subtypes (A–D, F–H, J, and K) with distinct phylogeny. Subtypes A and F can be further divided into sub-subtypes A1–A4 and F1 and F2, respectively. A number of inter-subtype recombinant viruses have also been observed [6–8].

HIV-1 group M subtypes are responsible for most of the HIV infections worldwide. In Italy the estimated percentage of non-B subtype infections has been reported to range from 2.4 to 19.4 %, thus confirming a significant increase in non-B subtypes prevalence [9–15], but, in this country, the first phase of the HIV epidemic was mainly confined to the intravenous drug users risk group, with an absolute predominance of HIV-1 B clade, as other Western Countries [16]. 

Phylogeny is a branch of molecular biology that infers knowledge about taxonomy and the evolution of species [17]. It is a powerful tool widely used in the study of rapidly evolving RNA viruses that cause chronic infections. The present case report underlines the importance of phylogenetic analysis to support epidemiological investigations into the reconstruction of transmission networks.

We present a case of HIV-1 subtype B transmission in a stable heterosexual couple living together was described to mark once more the importance of the “*tightened collaboration*” between phylogeny and epidemiology.

## Case presentation

We describe a case of HIV-1 subtype B transmission in a stable Caucasian heterosexual couple. The man was 30 years old and the woman was 21 years old at the time of their presentation to the Department of Public Health and Infectious Diseases of the University of Rome “Sapienza”. He became addicted to injected drugs in 1996 at the age of 14 and entered a rehabilitation community after 14 years of drug abuse, during which he practiced needle exchange. She was addicted to injected drugs from the age of 12 until the age of 19 and also practiced an unsafe needle exchange. They met after she joined his rehabilitation community.

Our epidemiological investigation was conducted in two different phases. At the beginning of April 2013, he came to our attention because of a suspected infection with hepatitis C virus (HCV). He reported a virulent and recent episode of shingles on his right hemi-thorax. During a physical examination we noticed the presence of several genital lesions, suggestive of condylomas. On the basis of his epidemiological history and these clinical findings, we proposed our patient be tested for HIV infection. He was found to be positive for HCV IgG (Anti HCV Advia Centaur Immunoassay System, Siemens Healthcare Diagnostic, Tarrytown, NY, USA) and negative for HCV RNA (Versant HCV RNA 1.0 assay (kPCR), Siemens Healthcare Diagnostics). He was HIV-Ag/Ab-positive (Advia Centaur Systems HIV Ag/Ab Combo assay, Siemens Healthcare Diagnostics) with an HIV RNA count of 102,900 copies/mL (Versant HIV 1.0 RNA assay (kPCR), Siemens Healthcare Diagnostics) and a CD4+ T cell count of 20 cells/μL (1.55 %). A genotypic resistance test (Trugene HIV-1 genotyping assay, Siemens Healthcare Diagnostics) showed a wild-type virus. Our patient started combination antiretroviral therapy (cART) with tenofovir, emtricitabine, atazanavir, and ritonavir. Owing to these findings, we tested our patient’s partner for HIV and HCV infections. She was HIV-Ag/Ab-negative, HCV IgG-positive, and HCV RNA-negative.

After two weeks, the woman presented to the emergency department of an urban hospital with an elevated temperature and a skin rash on every part of her body. Results of a blood test showed a white blood cell count of 1200 cell/mL. She was discharged with a diagnosis of a viral infection and instructed to present for ambulatory care to an infectious diseases clinic.

The day after this discharge, our patient arrived at our center and reported that her menses was two days late. She tested positive for beta-human chorionic gonadotropin. The test for HIV-Ag/Ab was repeated, again with negative results, but we tested her anyway for HIV RNA with a result of 4161 copies/mL. As stated by the US Department of Health and Human Services guidelines, in consideration of <10,000 copies/mL of HIV RNA with a negative HIV-Ag/Ab test, we repeated the HIV RNA test using a different specimen from the same patient, and found an HIV RNA count of 1,302,000 copies/mL. At this time, an HIV-Ag/Ab test had “undetermined” results, with a single gp41 positive band on a confirmatory western blot test. cART was initiated with tenofovir, emtricitabine, and raltegravir. As was the case with her partner, a genotypic resistance test revealed a wild-type virus. A genotyping test revealed coincident viral tropism within the couple, with CXCR4 tropism as predicted by a geno2pheno algorithm set at a false-positive rate of 10 % [18]. At the beginning of May, our patient had a spontaneous abortion due to acute retroviral syndrome.

To support the epidemiological investigation, we reconstructed the transmission network within a calendar timescale on the basis of a recently described phylogenetic–statistical framework, using the env viral sequences from our two patients [19, 20]. For virologic and phylogenetic analysis, we performed peripheral blood mononuclear cell isolation and DNA extraction as previously described [21]. The env region was amplified by a nested polymerase chain reaction and the following primers were used for the sequencing reaction: 5′-CTGTTAAATGGCAGTCTAGC-3′, 5′-GCAATGTATGCCCCTCCCATC-3′, and 5′GCTCCATGTTTTTCCAGGTC-3′. Sequence analyses were performed by Sequencher and Bioedit software packages. The subtypes of the two sequences was determined by uploading the sequences individually into the REGA HIV-1 automated Subtyping Tool v2.0 [22].

We built two different datasets: one with both the male and female sequences, and one without the female sequence to date the male infection. Nucleotide sequences were aligned with 35 reference HIV-1 subtype B sequences of known provenance and date (26 from Italy and six from other countries) using CLUSTAL W software and edited manually according to their codon-reading frame by BioEdit [23, 24]. These reference sequences were obtained with the Blast similarity search.

We performed a hierarchical likelihood ratio test using ModelTest 3.7 implemented in the PAUP* 4.0 software [23, 24], and identified the evolutionary model as the best-fitting nucleotide substitution model.

Dated phylogenies were obtained by simultaneously inferring the evolutionary rate and population and model parameters using a Bayesian Markov Chain Monte Carlo (MCMC) method implemented in the BEAST package version 1.8 [18, 25]. Statistical support for specific clades was obtained by calculating the posterior probability of each monophyletic clade. The trees were generated using the HKY +I+G model of substitution, chosen by ModelTest.

The MCMC was run for 50×10^6^ generations, under both strict and relaxed clock conditions, until convergence was achieved on the basis of the effective sampling size (ESS). Only ESS values of >250 were accepted. As coalescent priors, we compared three parametric models (constant, exponential, expansion growth) and a Bayesian Skyline plot non-parametric model.

For the first dataset, the expansion growth model under a relaxed (uncorrelated log normal) clock was selected, whereas for the second dataset, the exponential growth model under a relaxed (uncorrelated log normal) clock was selected.

**Fig. 1:**
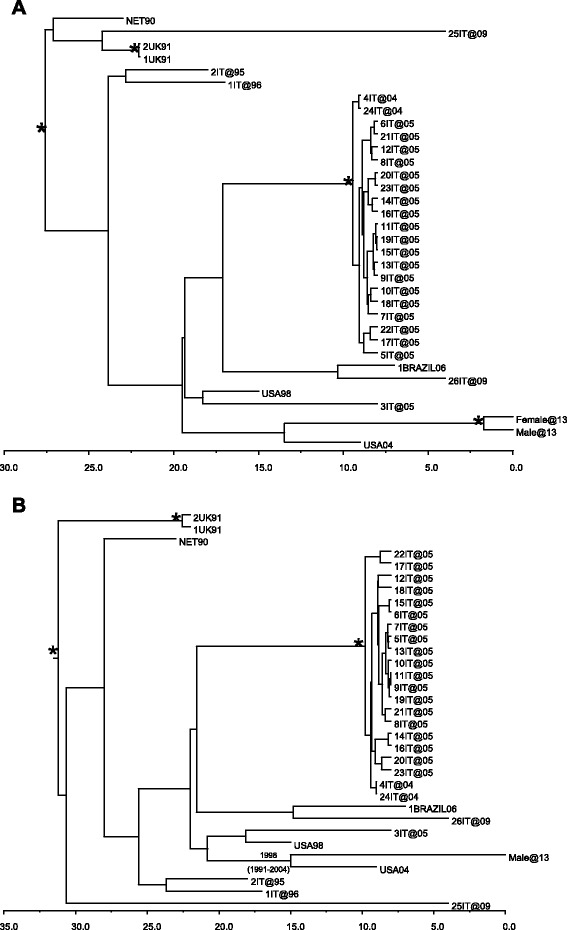
Bayesian time-scaled tree of the HIV-1 B sequences. The asterisks (*) along a branch represent significant statistical support for the clade subtending that branch (posterior probability = 100 %). The numbers at the internal node represent the estimated date of the origin and the uncertainty indicated by 95% highest posterior density intervals. **a** Couple (male and female) tree. **b** Dated male viral phylogeny

REGA subtyping analysis classified the two sequences as subtype B. Our patients’ isolates formed a significant monophyletic cluster (posterior probability = 1) (Fig. 1a), showing a strong relationship and affirming infection by the same virus.

In the male dated phylogeny (Fig. 1b), the male sequence was related to a sequence from the USA, and the time to most recent common ancestor was estimated to be 1998 (95 % highest posterior density, 1991 to 2004).

The Bayesian analysis confirmed the transmission network and allowed us to date the transmission event with a probability of 99 %. Moreover, the existence of an epidemiological relationship between the two patients confirmed the phylogenetic analysis and agreed with the presumptive date of infection on the basis of clinical history-taking. The Bayesian analysis also confirmed that our male patient probably acquired the infection about two years after starting illicit drug use and before having a relationship with our female patient.

## Conclusions

HIV-1 and HIV-2 transmission networks are already described in nosocomial and non-hospital-acquired infections [19, 20, 26]. A report of healthcare workers infection with HIV-1 by a needle stick injury was recently reported and published in 2010 [23]. A connection between epidemiological investigations and phylogenetic analyses was also recently demonstrated in case report analyses and population studies [24, 26]. Prospective surveillance studies conducted throughout the world report that the risk of HIV transmission ranges from 0.09 to 0.3 % [27].

A phylodynamic reconstruction, created using Bayesian methods, of the transmission network within a calendar timescale, that agreed with the epidemiological data, provided a well-documented transmission framework to significantly improve the investigation in our case.

Phylogenetic analysis is a powerful technique that if properly used can prove valuable in research investigations. In our case, we found it remarkable how the phylogenetic analysis and epidemiological evidence aligned to allow us to determine the most probable source of HIV infection. Our findings in these cases has strengthened the evidence that Bayesian phylogenetic analysis can be an important way of tracing epidemiological relationships.

## Consent

Written informed consent was obtained from the patients for publication of this case report and any accompanying images. A copy of the written consent is available for review by the Editor-in-Chief of this journal.

## Abbreviations

cART: combined antiretroviral therapy
ESS: effective sampling size
HCV: hepatitis C virus
HIV: human immunodeficiency virus
MCMC: Markov Chain Monte Carlo
